# Presumed atypical cor triatriatum dexter in a geriatric dog: A case report

**DOI:** 10.17221/50/2025-VETMED

**Published:** 2026-01-26

**Authors:** Yijin Jeong, Danbee Kwon, Kanghyo Park, Kichang Lee, Hakyoung Yoon

**Affiliations:** ^1^Department of Veterinary Medical Imaging, College of Veterinary Medicine, Jeonbuk National University, Iksan, Republic of Korea; ^2^Bundang Leaders Animal Medical Centre, Seongnam, Republic of Korea; ^3^Biosafety Research Institute and College of Veterinary Medicine, Jeonbuk National University, Iksan, Republic of Korea

**Keywords:** computed tomography, congenital heart disease, echocardiography, right sinus venosus valve remnants

## Abstract

Membranous structures within the right atrium (RA), such as the Chiari network, eustachian valve, Thebesian valve, and cor triatriatum dexter (CTD), can present with overlapping imaging features and complicated diagnoses. A 17-year-old male Maltese presented with a cardiac murmur, cough, anorexia, and exercise intolerance. Echocardiography revealed a mobile membrane in the RA, dividing it into two chambers. Computed tomography (CT) confirmed this finding, demonstrating differential attenuation of contrast between the chambers. The vascular connections and anatomical relationships of the structure differed from those of previously reported CTD types. Although other sinus venosus valve remnants were considered, the findings supported a provisional diagnosis of atypical CTD. This case highlights the utility of multimodal imaging for the characterisation of right atrial membranous structures. In human medicine, a detailed anatomical comparison of sinus venosus valve remnants aids in the differential diagnosis. Applying a similar approach in veterinary medicine, especially in geriatric patients, may improve diagnostic accuracy. Comprehensive imaging evaluations, including echocardiography and CT, are recommended to assess RA membranes that may be misinterpreted as variants of the CTD or other embryological remnants.

The right sinus venosus valve plays a critical role in foetal circulation by partitioning the right atrial lumen into two chambers and directing oxygenated blood from the caudal vena cava (CdVC) toward the foramen ovale for passage into the left atrium (LA). This mechanism facilitates perfusion of the head, neck, and forelimbs by bypassing the right ventricle (RV) and lung, thereby ensuring adequate oxygenation of the foetal brain (Kadiyala et al. 2021). After birth, the right sinus venosus valve gradually regresses, with its cranial portion forming the crista terminalis, and its caudal remnants contributing to the Eustachian valve (EV) at the ostium of the CdVC and the Thebesian valve (ThV) at the coronary sinus. However, incomplete regression may result in persistent remnants, such as the cor triatriatum dexter (CTD), Chiari network (CN), prominent EV, or prominent ThV. These residual structures can affect haemodynamic function depending on their size and location (Moral et al. 2016).

CTD, a rare congenital cardiac malformation in dogs, is characterised by the division of the right atrium (RA) into two chambers by a fibrous membrane, a remnant of the right sinus venosus valve (Jevens et al. 1993; Nadolny et al. 2019). Depending on its location and size, the membrane may obstruct venous return. These can be classified as perforate or imperforate, with the former being more common (Dobak et al. 2017; Nadolny et al. 2019). Anatomically, the cranial vena cava (CrVC) typically drains into the true RA connected to the RV, whereas the CdVC and coronary sinus enter the accessory RA (Adin and Thomas 1999; Mitten et al. 2001; Johnson et al. 2004; De Monte et al. 2015; Dobak et al. 2017; Nadolny et al. 2019; Phillips et al. 2021; Partington et al. 2022). Various congenital cardiac abnormalities are associated with CTD, including tricuspid valve dysplasia, pulmonic stenosis, patent foramen ovale, mitral valve dysplasia, double-chambered right ventricle, and patent ductus arteriosus (Nadolny et al. 2019). Although CTD has been reported in dogs aged two months to 12 years, it is mainly diagnosed in young dogs and is rare in geriatric dogs (Adin and Thomas 1999; Duncan et al. 1999; Mitten et al. 2001; Johnson et al. 2004; Arndt and Oyama 2008; Choi et al. 2014; De Monte et al. 2015; Dobak et al. 2017; Nadolny et al. 2019; Phillips et al. 2021; Partington et al. 2022). Clinical signs primarily include ascites and abdominal distension from right-sided congestive heart failure, as well as non-specific symptoms such as anorexia and exercise intolerance (Choi et al. 2014; Dobak et al. 2017; Nadolny et al. 2019; Phillips et al. 2021). Dilation and pulsation of the jugular vein have also been reported when the venous return from the CrVC is impaired (Partington et al. 2022). Although case reports of CTD are relatively common, reports describing other remnants of the right sinus venosus valve in veterinary medicine are scarce. Here, we describe a presumed atypical CTD characterised by a distinct anatomical structure that differs from previously reported cases and provide a comparative analysis of other right sinus venosus valve remnants that should be considered in the differential diagnosis of membranous structures within the RA.

## Case report

A 17-year-old intact male Maltese weighing 2.4 kg was referred to the Bundang Leaders Animal Medical Centre with a cardiac murmur and cough. Generalised cardiomegaly and multiple pulmonary nodules were identified on the previous thoracic radiographs. The patient was referred for echocardiography to assess cardiac function and computed tomography (CT) to evaluate possible primary tumours. The dog had an intermittent cough for a month, decreased appetite, and exercise intolerance.

On physical examination, the body condition score was 3/9, and no abdominal distention was noted. Tachycardia (195 beats/min) and a left apical systolic murmur (Grade IV/VI) were detected. Tachypnoea (56 breaths/min) was observed without abnormal lung sounds. No jugular vein distention or pulsation was observed.

A complete blood count revealed mild anaemia (haematocrit, 35.6%; reference range, 37–55%) and thrombocytopenia (152 × 10^9^ cells/l; reference range, 200–500 × 10^9^ cells/l). Serum biochemistry revealed elevated levels of alkaline phosphatase (335 U/l; reference range: 47–254 U/l), alanine transferase (150 U/l; reference range: 17–78 U/l), aspartate transferase (45 U/l; reference range: 17–44 U/l), and gamma-glutamyl transpeptidase (17 U/l; reference range: 5–14 U/l).

**Figure 1:**
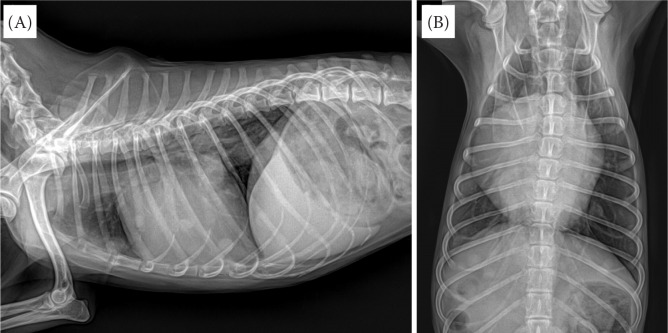
Thoracic radiography with right lateral (A) and ventrodorsal view (B) Generalised cardiomegaly and bulges in the regions of the right atrium and left atrial auricle are observed. Multiple soft-tissue opacity nodules are present in the lung field, and a mild interstitial pattern is observed in the right middle and caudal lung lobes

Thoracic radiography (HF99 525 Plus VET; EcoRay, Seoul, Republic of Korea; 58 kVp, 250 mA) revealed generalised cardiomegaly in the right lateral view (Figure 1A). In the ventrodorsal view, generalised cardiomegaly and bulges were observed at the 9–11 and 2–3 o’clock positions of the cardiac silhouette (Figure 1B). Multiple pulmonary nodules and mild pulmonary interstitial infiltration were observed in the right middle and caudal lobes (Figure 1). Distention of the CdVC was not significant. Abdominal radiography revealed generalised hepatomegaly and intact serosal details without abdominal distention.

**Figure 2:**
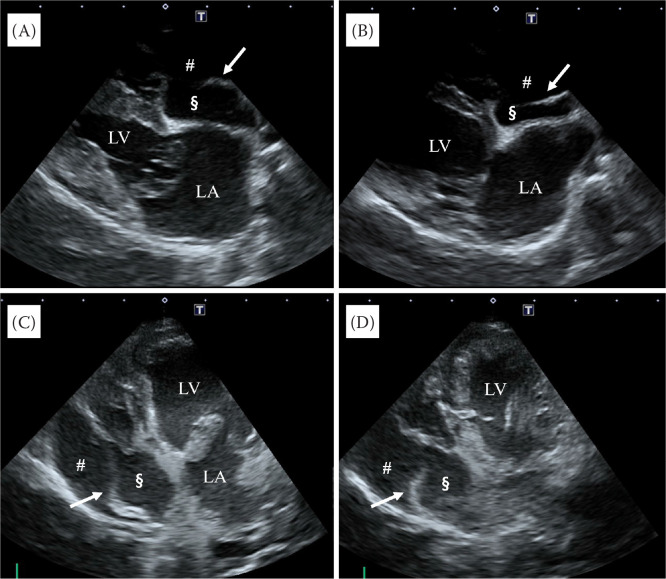
Transthoracic echocardiographic images of the right parasternal long-axis 4-chamber view (A,B) and left apical 4-chamber oblique view (C,D) In the right atrial region, a membranous structure (white arrow) is observed, dividing the right atrium into the proximal chamber (**§**) and distal chamber (#). Thickening of the mitral valve is observed (A,C)

Transthoracic echocardiography (Aplio 500; Canon Medical Systems, Ohtawara, Japan) using a 3.0 MHz sector transducer was performed to further evaluate cardiomegaly and cardiac murmurs. Marked thickening of the mitral valve and enlargement of the LA were observed on the right parasternal long-axis four-chamber view (Figure 2A). Degenerative changes in the tricuspid valve were also observed in the right parasternal short-axis view at the level of the pulmonary artery and in the left apical four-chamber view. During scanning, a mobile fibrous membranous structure dividing the chambers was identified in the RA in both the right parasternal long-axis and left apical four-chamber views (Figure 2). The perforated hyperechoic membrane divided the RA into a cranial proximal chamber (true RA), which contained the tricuspid orifice, and a caudal distal chamber (accessory RA) in the left apical four-chamber oblique view (Figure 2C,D). Concurrent cardiac anomalies were not observed.

**Figure 3:**
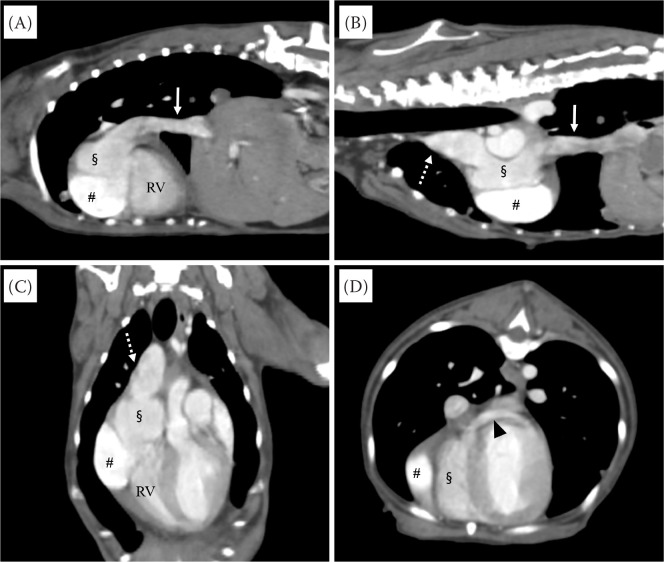
Reconstructed computed tomography images of the thorax Parasagittal (A,B), dorsal (c), and transverse plane (D). Entrances of the CrVC (white dashed arrow) and CdVC (white solid arrow) into the proximal chamber (**§**), which communicates with the RV, are observed. The hyperattenuating distal chamber (#) was separated from the proximal chamber by the fibrous membrane. The right coronary sinus (black arrowhead) is observed to connect to the proximal chamber (D) CrVC = cranial vena cava; RV = right ventricle

Whole-body CT (Brivo CT 385; GE Healthcare, Waukesha, WI, USA) was performed under general anaesthesia. The dog was premedicated with butorphanol (0.2 mg/kg i.v., Butophan Inj; Myungmoon Pharm, Hwaseong, Republic of Korea) and midazolam (0.1 mg/kg i.v.). Anaesthesia was induced using propofol (8 mg/kg i.v., Probive Inj.; Myungmoon Pharm, Hwaseong, Republic of Korea) and maintained with isoflurane (Ifran Liq; Hana Pharm, Hwaseong, Republic of Korea). Images with a 1.3 mm slice thickness at 120 kVp and 69 mA were acquired before and 1 min after the injection of iohexol contrast (2.5 ml/kg i.v., Omnipaque 300; GE Healthcare, Shanghai, P.R. China) into the right lateral saphenous vein. On the CT image of the heart, a nonselective venous angiogram revealed a membranous structure dividing the RA into two chambers, consistent with the echocardiographic findings. The proximal chamber communicated with the RV, and the entrances of the CrVC, CdVC, and coronary sinus were observed (Figure 3). The distal chamber had no vascular entrances. The flow of contrast medium confirmed communication between the two chambers (Figure 3A,C); however, the mean Hounsfield unit (HU) values differed. The mean HU of the accessory RA (381.4 ± 9.3) was 110 HU higher than that of the true RA (275.1 ± 8.6), while the true RA and RV (259.1 ± 11.6) were similar. The accessory RA had slightly higher mean HU values than the LA (325.9 ± 11) and left ventricle (LV) (335.9 ± 18.7). These differences in attenuation indicated partial compartmentalisation. Observation of contrast medium entering the accessory RA confirmed communication, suggesting that the membrane structure was fenestrated. No collateral vessels were observed.

Additional CT findings included multiple hypoattenuating hepatic nodules with rim enhancement and invasion of the adjacent CdVC. No ascites was detected. Multiple hyperattenuating pulmonary lesions were observed. Aside from the hepatic nodules, nodules in other organs were considered benign. Cytological or histological evaluations of the nodules were not performed; however, malignancy of the hepatic nodules with pulmonary metastasis was suspected. Owing to the dog’s geriatric age and presumed metastasis, he was discharged with medications for heart conditions, including pimobendan (0.25 mg/kg), furosemide (0.5 mg/kg), and sildenafil (1 mg/kg), twice daily. Two weeks later, thoracic radiography revealed recurrence of cardiogenic pulmonary oedema. The owner declined further testing, and the patient did not return for follow-up evaluation.

## DISCUSSION

Remnants of the right sinus venosus valve, including the CTD, CN, prominent EV, and prominent ThV, have distinct anatomical and clinical characteristics (Moral et al. 2016). A large membrane dividing the RA and the CTD may cause venous obstruction and right-sided heart failure. However, when the membrane is large and fenestrated, allowing sufficient blood flow, the CTD may remain asymptomatic and be incidentally detected in both humans and dogs (Arndt and Oyama 2008; Bindra et al. 2019; Nadolny et al. 2019). In contrast, the CN is a highly mobile, reticulated structure that rarely obstructs blood flow but may cause embolism or arrhythmias (Schneider et al. 1995; Loukas et al. 2010). The EV, located at the CdVC orifice, varies in size and shape; when prominent, it can appear as a linear membranous structure resembling CTD and CN (Yavuz et al. 2002; Watson et al. 2007). The ThV, a small fibrous remnant at the coronary sinus, has minimal haemodynamic impact and is rarely visible on transthoracic echocardiography but can be identified on transoesophageal echocardiography (Damodaran et al. 2021). Given their overlapping imaging features, differentiating them using echocardiography and CT is essential for an accurate diagnosis.

In the present case, a well-defined membranous structure traversing the RA was observed on echocardiography and CT. Compartmentalisation within the RA was supported by the differences in contrast attenuation between the two chambers. A prominent EV originating from a CdVC orifice typically has a short residual structure. Although giant EVs may appear elongated, they do not form compartments (Watson et al. 2007). Additionally, CT did not indicate its origin in the CdVC orifice, making this diagnosis unlikely. The CN can also be present as a membranous structure extending across the RA; however, it is typically thin and reticulated (Loukas et al. 2010), unlike the thicker, well-defined structure observed in this case. Finally, the prominent ThV was too small to be detected using transthoracic echocardiography, and the observed membranous structure did not originate from the coronary sinus orifice. Based on these differentiations, the structure in this case was tentatively diagnosed as non-obstructive CTD.

**Figure 4:**
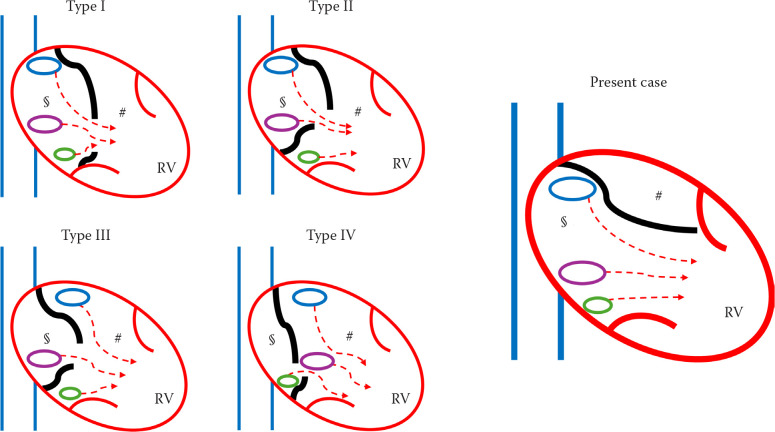
Schematic illustration of four previously described CTD subtypes and the present case To emphasise vascular connections between the two RA chambers, left-heart structures are omitted. The bold red line indicates the right heart margin and tricuspid valve, and the bold black curved line represents the membranous partition of the RA. The top blue (CrVC), bottom purple (CdVC), and small green (coronary sinus) circles mark venous openings, and thin dashed red arrows denote blood flow direction. In this case, the CrVC, CdVC, and coronary sinus open into the proximal chamber (**§**), with no vascular communication to the distal chamber (#), similar to Type I. Furthermore, the RV connects to the proximal chamber rather than the distal chamber, as in this case. The diagram was redrawn and modified based on anatomical descriptions from Hausdorf et al. (1985) and Partington et al. (2022) to highlight morphological variations and atypical venous inflow patterns in CTD CdVC = caudal vena cava; CrVC = cranial vena cava; CTD = cor triatriatum dexter; RA = right atrium; RV = right ventricle

The four morphological types of CTD in humans are classified according to the location of the membrane relative to the venous entrance into the RA (Doucette and Knoblich 1963; Hausdorf et al. 1985). Most canine cases are analogous to Type III, where the CrVC enters the distal chamber based on the membrane, whereas the CdVC enters the proximal chamber. In a typical CTD, the tricuspid valve and the RV are connected to a distal chamber. A previously reported CTD case was analogous to Type II and was described as an uncommon morphological variant in which both the CrVC and CdVC entered the proximal chamber (Partington et al. 2022). In this case, the CrVC, CdVC, and coronary sinus were connected to the proximal chamber, as in Type I. However, unlike typical Type I, the RV was also connected to the proximal chamber rather than the distal chamber. Thus, this case was considered an atypical form of CTD that did not fully align with Type I or other established types (Figure 4). Based on this anatomical difference, it was concluded that the fibrous membrane did not cause haemodynamic compromise, and no CTD-related clinical signs were evident in this case. The opening of the tricuspid valve into the proximal chamber was suspected to have resulted from pronounced remnants of the crista terminalis region, whereas the EV and ThV remnants appeared faint.

At diagnosis, 63% of canine CTD cases are under one year old, and only 13% are over five years (Adin and Thomas 1999; Duncan et al. 1999; Mitten et al. 2001; Johnson et al. 2004; Arndt and Oyama 2008; Choi et al. 2014; De Monte et al. 2015; Dobak et al. 2017; Nadolny et al. 2019; Phillips et al. 2021; Partington et al. 2022). Diagnosis in geriatric dogs is rare, with only one previous report of a 12-year-old mixed-breed dog (Arndt and Oyama 2008). Similarly, in the present case, the dog had a perforated membrane with sufficient blood flow but no clinical signs of right-sided congestive heart failure. The dog in this study, at 17 years old, represents the oldest reported case to date. Most affected dogs present with peritoneal effusion and abdominal distension, although 41% of the cases (7/17 dogs) are incidental findings in asymptomatic patients (Nadolny et al. 2019). Similarly, CTD in this case minimally contributed to the clinical signs and was diagnosed incidentally. In humans, CTD accounts for 0.1% of all congenital cardiac malformations and is mainly diagnosed in infants and children, with incidental findings in adults (Bindra et al. 2019). Incidental adult cases typically involve large fenestrations without clinical significance, supporting the hypothesis that perforated CTD remains haemodynamically insignificant.

Echocardiography and CT played complementary roles in the differential diagnosis. Echocardiography identified a movable membranous structure, aiding in differentiation from a right atrial diverticulum, which was difficult to distinguish on CT alone. A previous case of a right atrial diverticulum in a dog described the key distinguishing feature of CTD as its origin in the right atrial wall with a neck-like structure (Park et al. 2017). In the present case, a right atrial diverticulum was ruled out because the accessory chamber did not originate from the atrial wall but was instead delineated by a membranous structure within the RA. CT enables the detailed anatomical evaluation of vascular structures, including the CrVC, CdVC, and coronary sinus, clarifying their spatial relationships with membranous structures. CT also excluded other sinus venosus valve remnants and enabled comparison with reported CTD types, revealing that the findings did not fit any established type. Cardiac CT has emerged as a valuable adjunct tool for evaluating cardiac morphology and function, particularly when echocardiographic assessments are limited or inconclusive. Although echocardiography remains the primary diagnostic tool because of its real-time imaging and accessibility, CT provides complementary anatomical details, including visualisation of the cardiac chambers, valves, major vessels, and pericardium. The clinical indications for cardiac CT include congenital anomalies, cardiac or paracardiac masses, and pericardial diseases (Bertolini and Angeloni 2017). However, routine use in veterinary practice remains limited by equipment and technical demands. These findings highlight the importance of multimodal imaging for differentiating right atrial anomalies.

In addition to echocardiography, echocontrast studies, and cardiac CT, real-time fluoroscopic angiography can help evaluate intracardiac structures such as the CTD or remnants of the right sinus venosus valve. Although fluoroscopic angiography is frequently used during balloon dilation therapies in CTD patients (Johnson et al. 2004; Partington et al. 2022), it also offers diagnostic advantages by enabling dynamic visualisation of intracardiac blood flow. However, its diagnostic use is limited in routine clinical settings due to the need for general anaesthesia and catheter placement. Accordingly, most of the reported veterinary CTD cases do not include fluoroscopic angiography as part of the diagnostic process.

This case report had three limitations. First, this report lacks preserved colour Doppler images or video clips documenting the intracardiac blood flow between the two right atrial chambers. Although colour Doppler echocardiography was performed at the time of examination, and interchamber communication was confirmed, the images were not archived because the primary purpose was metastatic evaluation rather than detailed cardiac assessment. Second, contrast echocardiography or dynamic cardiac CT was not performed to confirm the dynamic flow between the chambers. However, evaluating HU values and contrast distribution on CT allows estimation of blood flow. Third, a postmortem examination was not performed; thus, a definitive diagnosis could not be established in comparison with conditions such as right sinus venosus valve remnants and right atrial diverticulum. This case was tentatively diagnosed as an atypical CTD, underscoring the need to consider various right sinus venosus valve remnants when assessing the membranous structures within the RA. Given the overlapping anatomical and imaging features, establishing a comprehensive differential diagnosis is essential. This case emphasises the value of multimodal imaging in assessing right atrial membranes and vascular relationships and highlights the need for differential diagnosis based on anatomical differences.
